# Correction: Kim et al. Effects of Nursing Simulation Using Mixed Reality: A Scoping Review. *Healthcare* 2021, *9*, 947

**DOI:** 10.3390/healthcare9121622

**Published:** 2021-11-24

**Authors:** Kyeng-Jin Kim, Moon-Ji Choi, Kyu-Jin Kim

**Affiliations:** 1 Department of Nursing, Kyungil University, Gyeongsan 38428, Korea; kkj0908@kiu.kr (K.-J.K.); cmoonj12@kiu.kr (M.-J.C.); 2 Daegu Center for Infectious Diseases Control and Prevention, Daegu 41940, Korea

The authors would like to make the following corrections about the published paper [1].

**Replacing the Institutional Review Board Statement from:** The study was approved by the Institutional Review Board of Kyung-il University (KIU-1041459-202007-HR-002-01).
